# Postresuscitation Management and Survival After Cardiac Arrest—The Whole Package

**DOI:** 10.1001/jamanetworkopen.2020.10921

**Published:** 2020-07-01

**Authors:** Joseph E. Tonna

**Affiliations:** Department of Surgery, University of Utah Health, Salt Lake City

Survival after cardiac arrest depends on the physician’s or medical team’s ability to restore perfusion to the heart during the cardiac arrest. This is often accomplished through administering medications, providing artificial respirations, and performing closed chest compressions. For decades, we have learned that early and effective cardiopulmonary resuscitation (CPR) and defibrillation are associated with increased probability of return of spontaneous circulation^1,2^; simultaneously, we have observed repeatedly that the probability of survival with intact neurologic function is inversely correlated with the duration of closed chest compressions before return of spontaneous circulation.^3,4^ Fundamentally, these observations suggest that, in most cases, CPR is suboptimal at providing perfusion to the organs, leading to progressive metabolic derangement and tissue ischemia.

However, physicians have a subtle advantage—myocardial cells have an inherent propensity to rhythmically contract. If physicians can provide sufficient coronary perfusion during CPR, notwithstanding a severe metabolic or hypoxic milieu or an acute coronary blockage, the heart will spontaneously beat, return of spontaneous circulation^1^ will be achieved, and the patient will have survived the cardiac arrest. In this issue, Girotra et al^5^ highlight that immediate cardiac arrest survival and overall survival to hospital discharge are often different for individual patients, with the latter outcome being worse.

Girotra et al^5^ present a retrospective cohort study using the American Heart Association (AHA) Get With The Guidelines–Resuscitation data set. Methodologically, the authors separated the survival rate of the cardiac arrest events from the postresuscitation hospital discharge survival rate. After risk standardization, they examined the correlation between each of these survival types with the overall survival to hospital discharge. The authors’ supposition was that these 2 survival rates were reflective of distinct phases of care—specifically, acute care during CPR vs postresuscitation care. There were 2 important principles underlying this analysis: (1) postresuscitation management and care was a distinct skill from resuscitation management and care, and (2) the quality of postresuscitation management and care had a measurable association with patient survival. Although randomized clinical trials have not clearly shown that these principles are true, behavior among physicians as though these principles are true is a major step toward improving resuscitation care.

Girotra et al^5^ showed a strong correlation between postresuscitation survival and overall hospital survival, a less strong correlation between event survival and hospital survival, and no significant correlation between event survival and postresuscitation survival. The study^5^ is important in that it provides data to emphasize the importance of postresuscitation management. The postcardiac arrest syndrome has been long recognized,^6^ but beyond the focus on therapeutic hypothermia or coronary catheterization after return of spontaneous circulation,^1^ there is less attention paid to variation in postresuscitation management and its association with outcomes. Girotra et al^5^ indicate that in the AHA data set, there are limited data available about features after return of spontaneous circulation^1^; therefore, it is difficult to understand their relative importance. Moreover, the authors also acknowledge that the AHA quality metrics focused largely on care during the cardiac arrest and less on the postresuscitation management phase.

One noteworthy study highlighting the importance of postresuscitation care by Tagami et al^7^ described a fifth link of care—the care after return of spontaneous circulation. In that study,^7^ the authors showed an adjusted association between comprehensive postresuscitation care and improved outcomes. This idea is showing support. Some centers are increasingly appreciating the importance of postresuscitation care and have implemented standard processes and postresuscitation care teams, such as the University of Pittsburgh Post Cardiac Arrest Service.^8^ As physicians, we must remember that from a physiologic perspective, the postresuscitation phase is a vulnerable one. In addition to experiencing tissue sensitivity from ischemia or reperfusion, other exposures happen to patients during this time. These exposures include receiving medications (with the possibility of medication errors), induced hypothermia and active temperature management, therapies for myocardial recovery, rehabilitation, and physical, occupational, or cognitive therapy.

In the spectrum of a hospital stay, the cardiac arrest event is an influential but short part. For more than a decade, the adoption of protocoled and prescribed resuscitation (ie, timed medication administration, scheduled pulse checks, high-quality chest compressions, and early defibrillation) has paralleled increased survival for in-hospital cardiac arrest. In contrast, the postresuscitation period and postresuscitation management has less prescribed behavior, and physiologic characteristics vary greatly. The postresuscitation period may be the longest part of the hospital stay and one that is—and needs to be—increasingly recognized as being associated with outcomes. Therefore, it is important to remember that although immediate cardiac arrest survival is necessary for long-term survival, it is not sufficient.

As researchers, we should direct our attention to the variation in care that occurs after cardiac arrest. Certain care components such as hyperoxia, induced hypothermia, coronary catheterization, and medication administration are known to be associated with outcomes, but even these are not fully characterized and more is unknown. Girotra et al^5^ showed a correlation between postresuscitation and overall hospital survival and that postresuscitation survival was correlated less strongly with survival after the cardiac arrest event. These findings suggest that as we once focused on improving cardiac arrest resuscitation through standardization, including the adoption of advanced cardiac life support, hospitals and physicians should now focus on improving postresuscitation intensive care through research and standard adoption of evidence-based practices. Participating in a successful resuscitation is a great feeling and wonderful for the patient, but we owe ourselves, our patients, and their families the opportunity to provide the same exceptional care throughout the entire postresuscitation care continuum.
